# Acute and subchronic toxicity assessment model of *Ferula assa-foetida* gum in rodents

**DOI:** 10.14202/vetworld.2015.584-589

**Published:** 2015-05-06

**Authors:** Ayman Goudah, Khaled Abdo-El-Sooud, Manal A. Yousef

**Affiliations:** Department of Pharmacology, Faculty of Veterinary Medicine, Cairo University, Giza, Egypt

**Keywords:** *F. assa-foetida*, histopathology, rats, serum enzymatic activity, subchronic toxicity

## Abstract

**Aim:**

The present study was performed to investigate acute and subchronic oral toxicity of *Ferula assa-foetida* gum (28 days) in Sprague Dawley rats.

**Materials and Methods:**

Acute oral administration of *F. assa-foetida* was done as a single bolus dose up to 5 g/kg in mice and subchronic toxicity study for 28 days was done by oral administration at doses of 0 (control) and 250 mg/kg in Sprague Dawley rats.

**Results:**

The obtained data revealed that oral administration of *F. assa-foetida* extract in rats for 28 successive days had no significant changes on body weight, body weight gain, the hematological parameters in rats all over the period of the experiment, and there are no significant increases in the activity of aspartate aminotransferase, alanine aminotransferase, alkaline phosphatase, creatinine and urea. Liver of treated rats showed mild changes as thrombosis and sinusoidal leukocytosis. It also showed portal infiltration with inflammatory cells, while kidney of treated rat showed an atrophy of glomerular tuft, thickening of parietal layer of Bowman capsule, and focal tubular necrosis. It also showed dilatation and congestion of renal blood vessels.

**Conclusion:**

We concluded that *F. assa-foetida* gum had broad safety and little toxicity for short term use in dose of 250 mg/kg.

## Introduction

Over the last two decades, the use of herbal remedies has expanded globally and traditional medicine has become very popular. According to the World Health Organization, about 80% of the world population, especially in developing countries, relies on plants for their health care [1].

From ancient times the medicinal properties of plants were discovered. Plants have been a constant source of drugs and recently, much emphasis has been placed on finding novel therapeutic agents from medicinal plants. Today many people prefer to use medicinal plants rather than chemical drugs [2]. Right from its beginning, the documentation of traditional knowledge especially on the medicinal uses of plants, has provided many important drugs of modern day [3].

The genus of *Ferula* belongs to the Peucedaneae tribe, a subfamily of Apioideae, family of Umbelliferae with 133 species distributed throughout the Mediterranean area and central Asia [4]. *Assa-foetida* is the oleo-gum-resin obtained from the roots and stems of many *Ferula* species; it is grayish white when fresh, darkening with age to yellow, red, and eventually brown. It is traditionally used for the treatment of different diseases, such as asthma, epilepsy, stomachache, flatulence, intestinal parasites, weak digestion, and influenza [5].

Recent pharmacological and biological studies have also shown several activities, such as antioxidant [6], antiviral [5], antifungal [7], anti-diabetic [8], molluscicidal [9], antispasmodic and hypotensive [10], and from this oleo-gum-resin.

*Assa-foetida* consists of three main fractions, including resin (40-64%), gum (25%), and essential oil (10-17%) [11]. The resin fraction contains ferulic acid and its esters, coumarins, sesquiterpene coumarins and other terpenoids. The gum includes glucose, galactose, L-arabinose, rhamnose, glucuronic acid, polysaccharides and glycoproteins, and the volatile fraction contains sulfur-containing compounds, monoterpenes, and other volatile terpenoids. Bioassay-guided fractionation studies of *assa-foetida* have led to the identification of some interesting bioactive compounds; for example, [5] characterized antiviral sesquiterpene coumarins from *assa-foetida* that are more potent than amantadine against influenza A.

Despite the wide use of this plant as food additive and herbal medicine in the treatment of a range of diseases, its toxicity is still unknown. Therefore, we evaluated the possible toxic effects of *Ferula assa-foetida* extract after acute and subchronic oral administration in rats.

## Materials and Methods

### Ethical approval

The use of the animals for the present study was reviewed and approved by the Institutional Animal Care and Use Committee at Faculty of Veterinary Medicine and maintained as per the guidelines of the committee for the purpose of control and supervision of experiments on animals.

### Plant

The *F*. *assa-foetida* extract (oleo-gum-resin), locally known as ‘‘ul-Heltit’’ of highest purity grade was purchased from a local market in Cairo, Egypt as solid brownish masses.

### Experimental animals

All animals (mature Swiss mice, 18-22 g b.wt and mature albino rats of Sprague Dawley strain, 140-170 g b.wt.) of both sexes were obtained from Laboratory Animal Colony, Helwan, Egypt. Animals were maintained in the Animal House of Pharmacology Department, Faculty of Veterinary Medicine, Cairo University under controlled hygienic conditions; received standard pellet ration and water was provided *ad-libitum*. The animals were subjected to a 12 h light and 12 h dark schedule and kept for 15 days before start of the experiment for acclimatization.

Immature albino rats of both sexes, 80-100 g b.wt. were used for studying the effect of prolonged administration of the extracts on body weight gain, blood criteria, activity of some serum enzymes, as well as, histopathological examination of liver and kidney samples.

### Acute oral toxicity

The oral acute toxicity test of *F*. *assa-foetida* extract was performed using [12] method. Three groups of 5 mice each were administered 10, 100, and 1000 mg/kg of extract orally. The mice were observed for 24 h for effects of toxicity and the number dying in each group within the period noted. When no deaths record, another four groups of 5 mice each were administered 2000, 3000, 4000, and 5000 mg/kg of extract orally. The animals were kept under observation for 48 h for effects of toxicity and the number dying in each group within the period were recorded. The LD_50_ value was calculated as the geometric mean of the highest non-lethal and the lowest lethal doses mathematically according to Kerber method [13] using the following formula:


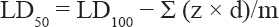


Where z is a half of sum of animal quantity died from two next doses; d is the interval between two next doses and m is the number of animals/group. LD_100_ is the largest dose which kills all animals.

### Subchronic oral toxicity

Healthy rats were randomly divided into two equal groups of 10 animals each. Group I (normal control) animals received 2% Tween 80 solution (0.5 ml, p.o.) and Group II animals received *F*. *assa-foetida* orally at 250 mg/kg dose. Animals were treated daily at 09 a.m by gavage for 28 days and were observed once daily to detect signs of toxicity. During the experimental period, the animals were weighed weekly and food intake and food conversion rates were monitored daily. At the end of the experiment, after 24 h of the last dose and 18 h fasting, animals were sacrificed, and blood samples were collected for hematological and biochemical parameters analysis. The liver and kidney were fixed in 10% formaldehyde for histological analysis. Paraffin sections of 5 µ thickness were prepared, stained by hematoxylene and eosin, and examined microscopically.

### Hematological studies

Blood samples were collected from retro-orbital of the experimental rats in capillary tubes coated with ethylenediaminetetraacetic acid. The tubes were immediately capped, kept at −4°C and were immediately analyzed for blood parameters using automated coagulating sysmex apparatus of the type 8999. The parameters included: Hemoglobin (Hb), mean cell volume (MCV), red blood cells count (RBCs), white blood cells count (WBCs), mean cell Hb concentration (MCHC), platelets (PLT), lymphocytes, and packed cell volume (PCV). However, MCV and MCHC values were calculated from RBCs count, Hb, and PCV [14].

### Biochemical estimation

Blood samples in dry tubes were centrifuged at 3500 rpm for 15 min and the supernatant (serum) was collected and introduced into new tubes and stored at −20°C until analyzed. The effect of *F*. *assa-foetida* treatment on the activity of serum aspartate aminotransferase (AST), serum alanine aminotransferase (ALT), serum alkaline phosphatase (ALP), creatinine, and urea were measured colorimetrically according to the method of [15-18], respectively, using (AST, ALT, ALP, creatinine, and urea) Biomerieux^®^ kits (BioMerieux Vitek, Inc., USA).

### Statistical analysis

The statistical analysis was performed using the SPSS^®^ 17.1 software package (SAS, Cary, NC, USA). Results are presented as mean ± standard error. The non-parametric Wilcoxon test was used to compare the parameters obtained.

## Results

### Acute oral toxicity

Acute toxicity studies on of *F*. *assa-foetida* extract were carried out on mice by oral administration. The safety of the *F*. *assa-foetida* extract is evidenced by the high LD_50_ value of the extract (>5 g/kg). In addition, there were no significant modification in the general behavior of the animals nor were there death after 72 h at the highest administered dose (5 g/kg) of the extract. Studies carried out to access the safety of this extract using mice revealed wide safety margin of the extract LD_50_>5 g/kg. The dose of 250 mg/kg of *F*. *assa-foetida* extract was chosen depending upon previous studies.

### Subchronic oral toxicity

Effect of prolonged oral administration of *F*. *assa-foetida* extract in rats for 28 successive days was investigated. In this respect, the effect on body weight gain, blood criteria, serum enzymatic activity, and changes histopathological figure on liver and kidneys was elucidated.

Oral administration of *F*. *assa-foetida* extract in rats for 28 successive days had no significant changes on body weight and body weight gain as summarized in Table-1.

**Table 1:** Effect of subchronic oral administration of *F. assa-foetida* (250 mg/kg) on body weight (g) of rats (n=10), (mean±SE)

Parameter	Control	*F. assa-foetida*
Initial weight (g)	111.43±6.52	109.71±4.62
Final weight (g)	148.57±4.11	149.41±6.52
Difference (g)	37.14±7.44	39.70±6.52
Difference (%)	33.33	36.19
Weight gained (g/day)	1.326	1.42

*F. assa-foetida*=*Ferula assa-foetida*, SE=Standard error

The effect of *F*. *assa-foetida* extract on blood parameters of rats after 28 days of daily oral administration was shown in Table-2. From the obtained data, it is clear that *F*. *assa-foetida* extract had no effect on the hematological parameters in rats all over the period of the experiment.

**Table 2:** Effect of subchronic oral administration of *F. assafoetida* (250 mg/kg) on hematological parameters of rats (n=10), (mean±SE)

Item	Control	*F. assa-foetida*
WBCs (×10^3^/mm^3^)	9.50±1.74	8.69±0.62
Lymphocytes (×10^3^/mm^3^)	8.54±1.59	7.30±0.58
Monocytes (×10^3^/mm^3^)	1.02±0.23	0.82±0.13
Granulocytes (×10^3^/mm^3^)	0.46±0.05	0.38±0.093
Lymph (%)	83.88±2.28	83.58±2.62
Monocytes (%)	10.78±1.57	8.86±1.00
Gran (%)	5.34±1.01	4.46±0.87
RBCs (×10^6^/mm^3^)	6.43±0.97	6.83±0.25
Hb (g/dl)	14.50±0.42	13.65±0.38
HCT (PCV) (%)	38.65±1.82	40.45±0.70
MCV (fl)	53.50±1.53	60.03±2.76
MCH (Pg)	19.24±0.23	20.34±1.21
MCHC (g/dl)	36.52±0.65	33.83±0.87
RDW-CV (%)	16.30±0.40	18.01±0.62
RDW-SD (fl)	39.00±3.40	44.93±5.38
PLT (×10^3^/mm^3^)	4.87±0.60	3.354±0.41
PCT (ml/l)	582.80±69.88	470.8±47.66
MPV (fl)	7.84±0.47	7.03±0.30
PDW (fl)	15.10±0.25	15.18±0.16
P-LCR (%)	21.50±4.11	16.57±2.34

*F. assa-foetida=Ferula assa-foetida*, SE=Standard error, WBCs=White blood cells, RBCs=Red blood cells, Hb=Hemoglobin, PCV=Packed cell volume, MCV=Mean cell volume, MCH=Mean cell hemoglobin, MCHC=Mean cell hemoglobin concentration, PLT=Platelets

The effect of prolonged oral administration of *F*. *assa-foetida* extract for 28 days on serum activity of AST, ALT, ALP, creatinine, and urea concentrations were recorded in Table-3. The obtained results showed that there are no significant increases in the activity of AST, ALT, ALP, creatinine, and urea in rats received *F*. *assa-foetida* extract as compared to control non treated group.

**Table 3:** Effect of subchronic oral administration of *F. assa-foetida* (250 mg/kg) on biochemical parameters of rats (n=10), (mean±SE)

Groups	ALT (IU/L)	AST (IU/L)	ALP (IU/L)	Urea (mg/dL)	Creatinine (mg/dL)
Control	28.2±1.36	175.40±30.74	273.8±24.11	42.7±2.11	0.68±0.08
*F. assa-foetida*	27.82±3.06	193.63±23.73	320.82±38.79	48.7±2.44	0.94±0.11

*F. assa-foetida*=*Ferula assa-foetida*, ALT=Alanine aminotransferase, AST=Aspartate aminotransferase, ALP=Alkaline phosphatase, SE=Standard error

**Figure-1:**
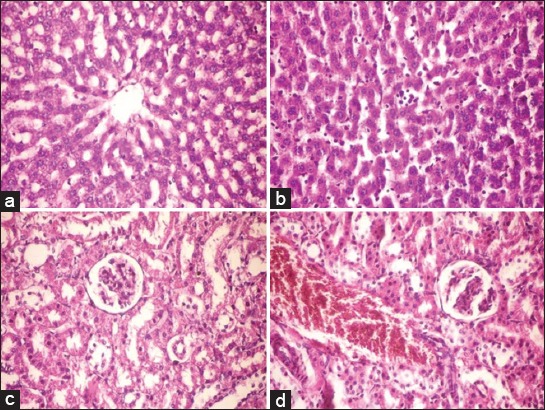
(a) Histopathological examination of liver, a section of liver tissue from a control and non-treated rat showed normal histological structure of hepatic lobule (hematoxylene and eosin. ×400), (b) histopathological examination of liver, a Section of liver of treated rat showed mild sinusoidal leukocytosis and congestion of blood sinusoids (hematoxylene and eosin. ×400), (c) histopathological examination of kidney, a Section of kidney tissue from a control and untreated rat showed the normal histological structure of renal parenchyma (hematoxylene and eosin. ×400), (d) histopathological examination of kidney, a section of kidney of treated rat showed dilatation and congestion of renal blood vessel (hematoxylene and eosin. ×400)

The histopathological findings of liver and kidneys of rats following the prolonged administration of *F*. *assa-foetida* for 28 days revealed that liver of treated rats showed mild changes as sinusoidal leukocytosis. It also showed portal infiltration with inflammatory cells (Figure-1a and b) as compared with control non treated group which showing normal histological structure of the hepatic lobule.

Kidney of treated rat showed a dilatation and congestion of renal blood vessels (Figure-1c and d) as compared with control non treated group which showed normal histological structure of normal parenchyma.

## Discussion

In the present study, acute and subchronic toxicity studies of *F*. *assa-foetida* extract were performed in order to evaluate the safety of this widely used medicinal plant.

Acute oral toxicity studies on *F*. *assa-foetida* extract revealed high LD_50_ value of the extract (>5 g/kg). In addition, there were no significant modification in the general behavior of the animals nor were there death after 72 h at the highest administered dose (5 g/kg) of the extract. Acute oral toxicity studies showed the lack of mortality and toxicity up to oral treatment of 5000 mg extract/kg body weight which suggests that the *F. assa-foetida* extract is practically non-toxic at single dose. However, in case of subsequent use in the treatment of the chronic diseases like diabetes or cancer, whether it will be safe that can be clear from its sub-chronic toxicity study.

Previous phytochemical investigation of the gum resin extract of *F. assa-foetida* was undertaken by way of an activity-guided fractionation procedure, which resulted in the isolation of galbanic acid (GBA) isolated from this herbal source, has antibiotic, anti-thrombotic, and hepatoprotective properties [19]. Another parallel study [20] suggests that GBA has strong anti-angiogenic activities and daily administration of GBA by intraperitoneal injection with as little as 1 mg/kg body weight can inhibit the growth of Lewis lung carcinoma allograft in syngeneic mice. Together with GBA, the related components karatavicinol, umbelliprenin, farnesiferol B, farnesiferol C, and GBA methyl ester belongs to sesquiterpene coumarin-containing compounds, some of which (such as the pyranocoumarin decursin) have been demonstrated to exert anti-androgen and antineoplastic activities [21,22]. Disulfides as well as symmetric tri- and tetrasulfides have been isolated [23] and the coumarin derivatives foetidin and kamolonol are also ­present [24]. However, plant- derived organic sulfides are subdivided structurally according to number of sulfur atoms and being linear or cyclic and bearing heteroatom [25]. In recent years, they have been assigned for various biological properties, including antioxidant, cancer chemopreventive, blood lipid reducing, having antibacterial, neuroprotective, and immune-modulatory effects in accordance with reported pharmacological effects of assafoetida.

In subchronic oral toxicity study, albino rats were used to test the nutritional values of *F. assa-foetida* via its effect on changes in the animal body weights. As results, no changes in body weight and no treatment-related changes in clinical signs were observed in rats suggesting that the 28 days treatment did not alter animal’s growth.

Hematological analysis showed that no significant modifications of the assessed parameters occurred in rats. According to a previous report, the hematopoietic system is one of the most sensitive targets for toxic substances [26]. However, mean values of each parameter were within the normal range [27]. There was no significant increase in the total WBCs count, PLT, monocytes, eosinophil, and basophil counts of the rats.

During the experimental period, there was no treatment related effect on Hb concentration and RBCs count which indicates the unlikelihood of the extract to induce anemia. Insignificant change in WBC count was probably due to normal response to foreign bodies or stress associated with the chronic toxicity studies [28].

Daily oral administration of *F. assa-foetida* for 28 days in rats induced no any significant changes of serum ALT, AST, ALP, urea, and creatinine activity. The normal level of AST, ALT, and ALP reflects the normal structural and functional functions of hepatic cells. The normal value of the hepatic biochemical parameters reveals the safety profile of the extract on liver function even on its chronic use.

ALP is a membrane bound enzyme while ALT and AST are cytosolic enzymes. These enzymes are highly concentrated in the liver and kidney and are only found in the serum in significant quantities when the cell membrane becomes leaky and even completely ruptured [29].

The fact that the levels of the enzymes were maintained in the liver and kidney in all groups of the rat means that the administered extract has no membrane labilizing effect on these organs. Enzyme activities in the tissues are often used as “marker” to ascertain early toxic effects of administered foreign compounds to experimental animals [30,31]. Since ALT is exclusively present in the cytoplasm of hepatocytes and AST mainly found in liver and heart [32]. Therefore, acute or chronic injury of the liver causes the elevation of their activities in the bloodstream [32,33]. On the other hand, although serum ALT activity is a highly sensitive biomarker of hepatotoxicity, slight elevations are often observed in rodents in the absence of correlative liver histomorphologic damages suggesting false positive or potentially prodromal signals [33]. In addition, damaged hepatocytes release both ALT and AST into the extracellular space. However, according to our results there were no differences in AST level in rats compared to control group suggesting no damage of hepatocytes. In addition, no changes were observed in serum creatinine and urea in rats. The serum creatinine level is a good indicator of renal function since any elevation of serum levels is associated to a marked failure of nephrons function [34]. Therefore, our results suggest that subchronic administration of *F. assa-foetida* did not induce hepatic venous thrombosis and did not alter renal function.

Examination of the *F. assa-foetida* treated rat kidney sections on 28^th^ day, in comparison to the control group, showed dilatation and congestion of renal blood vessels as compared with control non treated group which showed normal histological structure of normal parenchyma.

Examination of the *F. assa-foetida* treated rat liver sections on 28^th^ day of treated rats showed sinusoidal leukocytosis with inflammatory cells as compared with control non treated group which showing normal histological structure of the hepatic lobules. These results suggest that administration of gum extract of *F. assa-foetida* to rats did not have any adverse effect on the liver and kidney functions in rats showing that the extract is not toxic to man.

To our knowledge, there is no comprehensive toxicological study of assafoetida. However, a case of methemoglobinemia has been reported after administration of *assa-foetida* in a 5 weeks old black male infant. The infant was admitted to the hospital 6 h after the onset of tachypnea, grunting, and cyanosis. Treatment was with intravenous methylene blue and the infant recovered [35]. The intake of larger dosages can lead to swelling of the lips, digestive complaints such as flatulence and diarrhea, discomfort and headache. Swelling of the genital organs has been observed following external administration on the abdomen. It is not recommended to be used during pregnancy [36]. There is a need to study the details acute toxicity, sub-acute toxicity, chronic toxicity, and safety profiling of *F. assa-foetida*.

## Conclusion

Neither single oral administration of 5000 mg/kg nor the repeated doses (250 mg/kg) for 28 days of *F. assafoetida* induces mortality and obvious toxicological signs in rats. We concluded the safety and little toxicity of this extract for short term use in dose of 250 mg/kg. Hence, it is necessary to ascertain the scientific principle for the therapeutic actions of this gum extract as it may serve as the source for effective drug in the global market. Thus, the data presented in this study are very useful for the utilization of *F. assa-foetida* as natural remedy.

## Authors’ Contributions

AG and KA planned, designed the study, analyzed the data, participated in draft and revision of the manuscript and read and approved the final manuscript. AG, KA and MAY have equally contributed in collection of the samples and test. All authors participated in draft and revision. All authors read and approved the final manuscript.

## References

[1] Ateba S.B, Simo R.V, Mbanya J.C, Krenn L, Njamen D (2014). Safety profile and gender specific differences of a methanol extract of Eriosema laurentii (Leguminosae) in acute and subchronic (28 days) oral toxicity studies in Wistar rats. Food Chem. Toxicol.

[2] Mahendra P, Bisht S (2012). *Ferula asafoetida*: Traditional uses and pharmacological activity. Pharmacogn. Rev.

[3] Fabricant D.S, Farnsworth N.R (2001). The value of plants used in traditional medicine for drug discovery. Environ. Health Persp.

[4] Zhang Y, Kim K.H, Zhang W, Guo Y, Kim S.H, Lü J (2012). Galbanic acid decreases androgen receptor abundance and signaling and induces G1 arrest in prostate cancer cells. Int. J. Cancer.

[5] Lee C.L, Chiang L.C, Cheng L.H, Liaw C.C, Abd El-Razek M.H, Chang F.R, Wu Y.C (2009). Influenza A (H1N1) Antiviral and Cytotoxic Agents from *Ferula assafoetida*. J. Nat. Prod.

[6] Nabavi S.M, Ebrahimzadeh M.A, Nabavi S.F, Eslami B, Dehpour A.A (2011). Antioxidant and antihaemolytic activities of *Ferula foetida* regel (Umbelliferae). Eur. Rev. Med. Pharmaco.

[7] Angelini P, Pagiotti R, Venanzoni R, Granetti B (2009). Antifungal and allelopathic effects of *asafoetida* against Trichoderma harzianum and Pleurotus spp. Allelopathy J.

[8] Abu-Zaiton A.S (2010). Anti-diabetic activity of *Ferula assafoetida* extract in normal and alloxan-induced diabetic rats. Pak. J. Biol. Sci.

[9] Kumar P, Singh D.K (2006). Molluscicidal activity of *Ferula asafoetida* Syzygium aromaticum and Carum carvi and their active components against the snail Lymnaea acuminata. Chemosphere.

[10] Fatehi M, Farifteh F, Fatehi-Hassanabad Z (2004). Antispasmodic and hypotensive effects of *Ferula asafoetida* gum extract. J. Ethnopharmacol.

[11] Abd El-Razek M.H, Ohta S, Ahmed A.A, Hirata T (2001). Sesquiterpene coumarins from the roots of *Ferula assafoetida*. Phytochemistry.

[12] Lorke D (1983). A new approach to practical acute toxicity testing. Arch. Toxicol.

[13] Pershin G.K (1971). Methods of experimental chemotherapy: Practical guidance.

[14] Merghani T.H (2010). The Core of Medical Physiology.

[15] Reitman S, Frankel S (1957). A colorimetric method for the determination of serum glutamic oxalacetic and glutamic pyruvic transaminases. Am J Clin. Pathol.

[16] Kroll M.H, Roach N.A, Poe B, Elin R.J (1987). Mechanism of interference with the Jaffe reaction for creatinine. Clin. Chem.

[17] Kind P.R, King E.J (1954). Estimation of plasma phosphatase by determination of hydrolysed phenol with amino-antipyrine. J. Clin. Pathol.

[18] Wills M.R, Savory J (1981). Biochemistry of renal failure. Ann. Clin. Lab. Sci.

[19] Mansurov M.M, Martirosov M.S (1990). The action of the sodium salt of galbanic acid in experimental erythrocyte hyperaggregation. Farmakol. Toksikol.

[20] Kim K.H, Lee H.J, Jeong S.J, Lee H.J, Lee E.O, Kim H.S, Zhang Y, Ryu S.Y, Lee M.H, Lu J, Kim S.H (2011). Galbanic acid isolated from *Ferula assafoetida* exerts in vivo anti-tumor activity in association with anti-angiogenesis and anti-proliferation. Pharmaceut. Res.

[21] Jiang C, Lee H.J, Li G.X, Guo J, Malewicz B, Zhao Y, Lee E.O, Lee H.J, Lee J.H, Kim M.S, Kim S.H, Lu J (2006). Potent antiandrogen and androgen receptor activities of an Angelica gigas-containing herbal formulation: Identification of decursin as a novel and active compound with implications for prevention and treatment of prostate cancer *Cancer Res* 66 (1).

[22] Guo J, Jiang C, Wang Z, Lee H.J, Hu H, Malewicz B, Lee H.J, Lee J.H, Baek N.I, Jeong J.H, Kim D.K, Kang K.S, Kim S.H, Lu J (2007). A novel class of pyranocoumarin anti-androgen receptor signaling compounds. Mol. Cancer. Ther.

[23] Rajanikanth B, Ravindranath B, Shankaranarayana M.L (1984). Volatile polysulphides of *asafoetida*. Phytochemistry.

[24] Hofer O, Widhalm M, Greper H (1984). Circular dichroism of sesquitergeneumbelliferone ethers and structure elucidation of a new derivative isolated from the gum resin 'assafoetida. Monatsh. Chem.

[25] Iranshahi M (2012). A review of volatile sulfur-containing compounds from terrestrial plants: Biosynthesis, distribution and analytical methods. J. Essent. Oil Res.

[26] Harper H.A (1973). Review of physiological chemistry.

[27] David K, Weber K, Danielson S, Wright J, Foley E (2002). Hematology and serum biochemistry values of rats. J. Wildlife Dis.

[28] Kelly (1974). Veterinary clinical diagnosis.

[29] Cotran R, Kumar V, Robins S (1989). Robin's pathological basis of disease.

[30] Adesokan A.A, Akanji M.A (2004). Effect of administration of aqueous extract of Enantia chlorantha on the activities of some enzymes in the small intestine of rats. Niger. J. Biochem. Mol. Biol.

[31] Ramaiah S.K (2007). A toxicologist guide to the diagnostic interpretation of hepatic biochemical parameters. Food and chemical toxicology. Food Chem. Toxicol.

[32] Han Y.D, Song S.Y, Lee J.H, Lee D.S, Yoon H.C (2011). Multienzyme-modified biosensing surface for the electrochemical analysis of aspartate transaminase and alanine transaminase in human plasma. Anal. Bioanal. Chem.

[33] Ozer J, Ratner M, Shaw M, Bailey W, Schomaker S (2008). The current state of serum biomarkers of hepatotoxicity. Toxicology.

[34] Lameire N. Van, Biesen W, Vanholder R (2005). Acute renal failure. Lancet.

[35] Kelly K.J, Neu J, Camitta B.M, Honig G.R (1984). Methemoglobinemia in an infant treated with the folk remedy glycerited *asafoetida*. Pediatrics.

[36] Emami A, Fasihi S, Mehregan I (2010). Medicinal Plants.

